# 3,3′-Dibenzoyl-1,1′-dibenzyl-1,1′-(ethane-1,2-di­yl)­dithio­urea

**DOI:** 10.1107/S1600536812002954

**Published:** 2012-02-04

**Authors:** Andrzej Okuniewski, Jaroslaw Chojnacki, Barbara Becker

**Affiliations:** a Department of Inorganic Chemistry, Gdansk University of Technology, 11/12 Narutowicza Street, 80-233 Gdańsk, Poland

## Abstract

In the title compound, C_32_H_30_N_4_O_2_S_2_, the carbonyl and thio­carbonyl groups are found in a rare synclinal conformation, with an S—C⋯C—O pseudo-torsion angle of 62.6 (2)°. The mol­ecule has *C_i_* = *S*_2_ point-group symmetry with a crystallographic center of inversion located in the middle of the ethyl­ene bridge. One of the symmetry-independent phenyl rings is disordered over two orientations, with a site-occupation ratio of 70:30. The distances between the centroids of the nearest phenyl rings are equal to one of the lattice constants [*a* = 4.7767 (2) Å], so stacking inter­actions are extremely weak. Mol­ecules are joined by bifurcated hydrogen bonds (N—H⋯O and N—H⋯S), forming a ladder-like arrangement along [100]. van der Waals forces combine these ladders into a three-dimensional structure. The dependency between the S⋯O distance and the improper S=C⋯C=O torsion angle based on 739 structures containing the CC(=O)NC(=S)N moiety is discussed.

## Related literature

For structures of bis­(*N*-benzoyl­thio­ureas) derived from aliphatic diamines, see: Ding *et al.* (2008 ▶); Dong *et al.* (2007 ▶); Sow *et al.* (2009 ▶). For those derived from *o*-cyclo­hexa­ne­diamine, see: Jumal *et al.* (2011 ▶). For those derived from aromatic diamines, see: Cao *et al.* (2007 ▶); Li *et al.* (2009 ▶); Thiam *et al.* (2008 ▶); Woei Hung & Kassim (2010 ▶); Yamin & Osman (2011 ▶). For other acyl derivaties obtained from *o*- and *p*-phenyl­enediamine (also solvates), see: Dong, Yan *et al.* (2008 ▶); Dong, Yang *et al.* (2008 ▶); Du & Du (2008 ▶); Du *et al.* (2008 ▶). For 1-benzoyl-3-phenyl­urea, see: Okuniewski *et al.* (2010 ▶). For the synthetic procedure, see: Douglass & Dains (1934 ▶). For a review on *N*-aroyl­thio­ureas, see: Aly *et al.* (2007 ▶). For a description of the Cambridge Structural Database, see: Allen (2002 ▶).
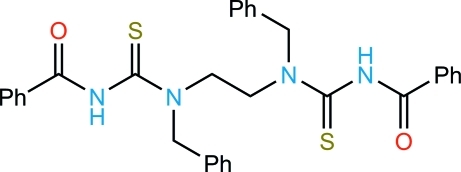


## Experimental

### 

#### Crystal data

C_32_H_30_N_4_O_2_S_2_
*M* *_r_* = 566.72
Monoclinic, 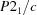

*a* = 4.7767 (2) Å
*b* = 25.1653 (16) Å
*c* = 11.9998 (8) Å
β = 91.585 (5)°
*V* = 1441.91 (15) Å^3^
*Z* = 2
Mo *K*α radiation
μ = 0.22 mm^−1^
*T* = 298 K
0.61 × 0.18 × 0.08 mm

#### Data collection

Oxford Diffraction Xcalibur diffractometer with a Sapphire2 (large Be window) detector
Absorption correction: multi-scan (*CrysAlis PRO*; Oxford Diffraction, 2010 ▶) *T*_min_ = 0.780, *T*_max_ = 1
7277 measured reflections
2685 independent reflections
2021 reflections with *I* > 2σ(*I*)
*R*_int_ = 0.031

#### Refinement

*R*[*F* ^2^ > 2σ(*F* ^2^)] = 0.05
*wR*(*F* ^2^) = 0.123
*S* = 1.04
2685 reflections
231 parameters
163 restraints
H atoms treated by a mixture of independent and constrained refinement
Δρ_max_ = 0.30 e Å^−3^
Δρ_min_ = −0.15 e Å^−3^

### 

Data collection: *CrysAlis PRO* (Oxford Diffraction, 2010 ▶); cell refinement: *CrysAlis PRO*; data reduction: *CrysAlis PRO*; program(s) used to solve structure: *SHELXS97* (Sheldrick, 2008 ▶); program(s) used to refine structure: *SHELXL97* (Sheldrick, 2008 ▶); molecular graphics: *OLEX2* (Dolomanov *et al.*, 2009 ▶); software used to prepare material for publication: *WinGX* (Farrugia, 1999 ▶) and *PLATON* (Spek, 2009 ▶).

## Supplementary Material

Crystal structure: contains datablock(s) global, I. DOI: 10.1107/S1600536812002954/im2351sup1.cif

Structure factors: contains datablock(s) I. DOI: 10.1107/S1600536812002954/im2351Isup2.hkl

Supplementary material file. DOI: 10.1107/S1600536812002954/im2351Isup3.smi

Supplementary material file. DOI: 10.1107/S1600536812002954/im2351Isup4.cml

Additional supplementary materials:  crystallographic information; 3D view; checkCIF report

## Figures and Tables

**Table 1:** Hydrogen-bond geometry (Å, °)

*D*—H⋯*A*	*D*—H	H⋯*A*	*D*⋯*A*	*D*—H⋯*A*
N1—H1N⋯O1^i^	0.86 (1)	2.30 (1)	3.073 (2)	150 (2)
N1—H1N⋯S1^i^	0.86 (1)	2.98 (2)	3.647 (2)	136 (2)

Symmetry code: (i) 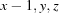.

**Fig. 1.**
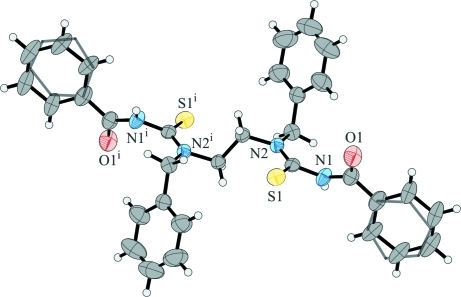
The molecular structure of the title compound with displacement ellipsoids drawn at the 50% probability level. Symmetry code: (i) –x + 2, –y + 1, –z + 1.

**Fig. 2.**
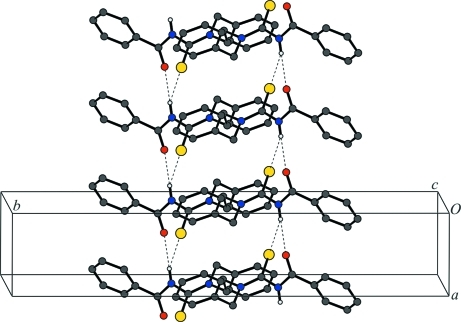
Ladder-like structural motif found in the structure of the title compound. Hydrogen bonds are shown as dashed lines. C-bonded hydrogen atoms have been ommited for clarity.

**Fig. 3.**
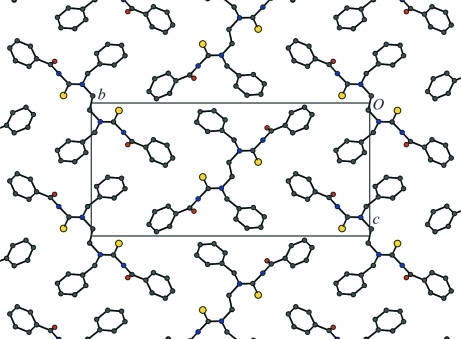
Tiling of ladders seen along [100] direction. Hydrogen atoms have been ommited for clarity.

**Fig. 4.**
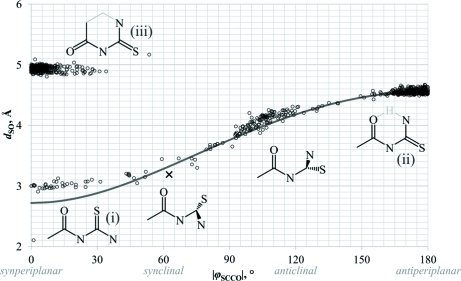
Plot of S···O distance (dSO) against absolute value of S=C···C=O torsion angle (|φSCCO|) for 739 structures containing CC(=O)NC(=S)N fragment found in CSD 5.32 (Allen, 2002). Title compound is marked with cross. Solid line represents theoretical relation (see: comment).

## supplementary crystallographic information

### Comment

Substituted *N*-acylthioureas are the subject of extensive research because of their biological activity, metal coordination ability and hydrogen bond formation (Aly *et al.*, 2007). Bis(*N*-acylthioureas) are a reatively less studied group with respect to their mono-analogues (Yamin & Osman, 2011).

The title compound, [PhCONHCSN(CH_2_Ph)CH_2_]_2_ (Fig.1), has the inversion center located in the middle of the ethylene bridge, so only half of the molecule is symetrically independent. There is no intramolecular N—H···O hydrogen bond that is commonly present in substituted *N*-acylthioureas and ureas (Okuniewski *et al.*, 2010), because the hydrogen atom on N2 is substituted by a benzyl group. The only specific interactions are weak bifurcative N—H···O and N—H···S intermolecular hydrogen bonds joining molecules into one-dimensional ladders along [100] (Fig. 2). The three-dimensional structure is only stabilized by van der Waals forces (Fig. 3).

Three main geometries of *N*-acylthioureas based on the S···O distance (*d*_SO_) and the S=C···C=O improper torsion angle (*φ*_SCCO_) can be distinguished: synperiplanar type (i) and (iii) with |*φ*_SCCO_ | ≈ 0° as well as antiperiplanar type (ii) with |*φ*_SCCO_ | ≈ 180° (Fig. 4). The S···O distance in type (i) is about 3 Å while in type (iii) is about 5 Å. Transition between type (i) and (ii) is smooth and is accomplished by rotation about the thioamide bond. Theoretical relation between *d*_SO_ and *φ*_SCCO_ assuming constant bond lengths and valence angles can be expressed as (see: solid line in Fig. 4):

*d*_SO_ = (*A* cos *φ*_SCCO_ + *B*)^0.5^

where *A* and *B* are calculated as:

*A* = 2 *d*_SC_ sin *α*_SCN_ [*d*_CO_ sin(*α*_NCO_ + *α*_CNC_) – *d*_NC_ sin *α*_CNC_] ≈ -6.4657 Å^2^

*B* = [–*d*_CN_ + *d*_CN_ cos *α*_CNC_ – *d*_CO_ cos(*α*_NCO_ + *α*_CNC_) + *d*_SC_ cos *α*_SCN_]^2^ + [–*d*_SC_ sin *α*_SCN_]^2^ + [*d*_NC_ sin *α*_CNC_ – *d*_CO_ sin(*α*_NCO_ + *α*_CNC_)]^2^≈ 13.666 Å^2^

Numerical values of bond lengths and angles are the average ones calculated in Vista program on the basis of 739 structures (980 values) containing CC(=O)NC(=S)N moiety found in CSD 5.32 (Allen, 2002)

Type (ii) is generally more stable than type (i) due to the formation of the intramolecular N—H···O hydrogen bond. When there is no suitable hydrogen atom to form hydrogen bonds (*N*,*N*-disubstituted derivatives) the anticlinal geometry (|*φ*_SCCO_ | ≈ 120°) is preferred. Only 58 out of 980 points in Fig. 4 represent type (i) with *d*_SO_ < 4 Å and |*φ*_SCCO_ | < 90°. In this type the S···O distance is slightly greater than theoretical value due to sulfur-oxygen repulsion. Molecules of type (iii) contain covalent six-membered rings (see: structure (iii) in Fig. 4) preventing any rotation, so there is no possibility to transform this type to any other.

Geometric parameters of the title compound's molecule place it near type (i) – rare synclinal conformation with |*φ*_SCCO_| = 62.60 (18)° (see: cross mark in Fig. 4). Large substituents on N2 atom cause type (ii) to be geometrically unfavourable.

### Experimental

Synthesis was performed according to Douglass & Dains (1934): 2.50 g (33 mmol) of ammonium thiocyanate and 20 ml of acetone were placed in a two-necked flask. Through a dropping funnel 3.49 ml (30 mmol) of benzoyl chloride in 20 ml of acetone was added with stirring. After addition was completed the mixture was refluxed for additional 15 min and then 3.54 ml (15 mmol) of *N*,*N'*-dibenzylethane-1,2-diamine in 20 ml of acetone was added through the dropping funnel. The mixture was carefully poured to the 500 ml of water with stirring. The resulting precipitate was filtered on a Büchner funnel. The crude product was recrystallized from acetone. Colorless single crystals suitable for X-ray diffraction analysis were isolated with 72% yield. Melting point: 165 (1)°C.

### Refinement

All C-bonded hydrogen atoms were placed in calculated positions (aromatic: *d*_CH_ = 0.97 Å, methylene: *d*_CH_ = 0.93 Å) and were treated as riding on their parent atoms with *U*_iso_ (H) = 1.2 *U*_eq_(C). H1*N* atom was located from difference Fourier map and refined isotropically with *d*_NH_ restrained to 0.88 (1) Å.

### Crystal data

**Table:**

C_32_H_30_N_4_O_2_S_2_	*F*(000) = 596
*M**_r_* = 566.72	*D*_x_ = 1.305 Mg m^−^^3^
Monoclinic, *P*2_1_/*c*	Melting point: 438(1) K
Hall symbol: -P 2ybc	Mo *K*α radiation, λ = 0.71073 Å
*a* = 4.7767 (2) Å	Cell parameters from 2955 reflections
*b* = 25.1653 (16) Å	θ = 2.3–28.7°
*c* = 11.9998 (8) Å	µ = 0.22 mm^−^^1^
β = 91.585 (5)°	*T* = 298 K
*V* = 1441.91 (15) Å^3^	Needle, colourless
*Z* = 2	0.61 × 0.18 × 0.08 mm

### Data collection

**Table:**

Oxford Diffraction Xcalibur Sapphire2 (large Be window) diffractometer	2685 independent reflections
Graphite monochromator	2021 reflections with *I* > 2σ(*I*)
Detector resolution: 8.1883 pixels mm^-1^	*R*_int_ = 0.031
ω scans	θ_max_ = 25.5°, θ_min_ = 2.4°
Absorption correction: multi-scan (*CrysAlis PRO*; Oxford Diffraction, 2010)	*h* = −5→5
*T*_min_ = 0.780, *T*_max_ = 1	*k* = −26→30
7277 measured reflections	*l* = −14→14

### Refinement

**Table:**

Refinement on *F*^2^	Primary atom site location: structure-invariant direct methods
Least-squares matrix: full	Secondary atom site location: difference Fourier map
*R*[*F*^2^ > 2σ(*F*^2^)] = 0.05	Hydrogen site location: inferred from neighbouring sites
*wR*(*F*^2^) = 0.123	H atoms treated by a mixture of independent and constrained refinement
*S* = 1.04	*w* = 1/[σ^2^(*F*_o_^2^) + (0.0551*P*)^2^ + 0.2801*P*] where *P* = (*F*_o_^2^ + 2*F*_c_^2^)/3
2685 reflections	(Δ/σ)_max_ = 0.016
231 parameters	Δρ_max_ = 0.30 e Å^−^^3^
163 restraints	Δρ_min_ = −0.15 e Å^−^^3^

### Special details

**Table:**

Experimental. CrysAlisPro, Oxford Diffraction Ltd., Version 1.171.33.66 (Oxford Diffraction, 2010) Empirical absorption correction using spherical harmonics, implemented in SCALE3 ABSPACK scaling algorithm.
Geometry. All e.s.d.'s (except the e.s.d. in the dihedral angle between two l.s. planes) are estimated using the full covariance matrix. The cell e.s.d.'s are taken into account individually in the estimation of e.s.d.'s in distances, angles and torsion angles; correlations between e.s.d.'s in cell parameters are only used when they are defined by crystal symmetry. An approximate (isotropic) treatment of cell e.s.d.'s is used for estimating e.s.d.'s involving l.s. planes.
Refinement. Refinement of *F*^2^ against ALL reflections. The weighted *R*-factor *wR* and goodness of fit *S* are based on *F*^2^, conventional *R*-factors *R* are based on *F*, with *F* set to zero for negative *F*^2^. The threshold expression of *F*^2^ > σ(*F*^2^) is used only for calculating *R*-factors(gt) *etc*. and is not relevant to the choice of reflections for refinement. *R*-factors based on *F*^2^ are statistically about twice as large as those based on *F*, and *R*- factors based on ALL data will be even larger.

### Fractional atomic coordinates and isotropic or equivalent isotropic displacement parameters (Å^2^)

**Table:**

	*x*	*y*	*z*	*U*_iso_*/*U*_eq_	Occ. (<1)
C1	1.1371 (4)	0.58001 (8)	0.64272 (17)	0.0356 (5)	
C3	1.0855 (4)	0.49446 (8)	0.55324 (16)	0.0377 (5)	
H3A	1.0468	0.4587	0.5788	0.045*	
H3B	1.2835	0.4967	0.5379	0.045*	
C10	1.2067 (5)	0.64605 (9)	0.7895 (2)	0.0458 (5)	
C11	1.0914 (5)	0.69614 (9)	0.8323 (2)	0.0528 (6)	
C12A	0.882 (3)	0.7252 (6)	0.7782 (14)	0.065 (3)	0.71 (3)
H12A	0.7981	0.713	0.7121	0.078*	0.71 (3)
C13A	0.800 (2)	0.7731 (4)	0.8256 (11)	0.077 (3)	0.71 (3)
H13A	0.6566	0.7927	0.7911	0.092*	0.71 (3)
C14A	0.924 (2)	0.7922 (3)	0.9218 (13)	0.078 (3)	0.71 (3)
H14A	0.8664	0.8244	0.9513	0.093*	0.71 (3)
C15A	1.138 (3)	0.7634 (4)	0.9756 (10)	0.078 (3)	0.71 (3)
H15A	1.2214	0.7762	1.0413	0.093*	0.71 (3)
C16A	1.225 (3)	0.7153 (5)	0.9299 (9)	0.065 (2)	0.71 (3)
H16A	1.3702	0.6961	0.9638	0.077*	0.71 (3)
C12B	0.873 (7)	0.7181 (15)	0.769 (3)	0.075 (7)	0.29 (3)
H12B	0.8008	0.6968	0.7118	0.091*	0.29 (3)
C13B	0.747 (7)	0.7674 (12)	0.780 (3)	0.097 (6)	0.29 (3)
H13B	0.6192	0.7814	0.7281	0.116*	0.29 (3)
C14B	0.831 (7)	0.7934 (10)	0.875 (2)	0.083 (6)	0.29 (3)
H14B	0.7374	0.8243	0.8941	0.099*	0.29 (3)
C15B	1.048 (7)	0.7760 (10)	0.943 (3)	0.080 (7)	0.29 (3)
H15B	1.1111	0.7964	1.0035	0.095*	0.29 (3)
C16B	1.168 (7)	0.7275 (11)	0.920 (2)	0.075 (7)	0.29 (3)
H16B	1.3107	0.7155	0.9683	0.09*	0.29 (3)
C20	0.8164 (4)	0.51283 (9)	0.72178 (18)	0.0410 (5)	
H20A	0.6511	0.4995	0.6822	0.049*	
H20B	0.7592	0.5421	0.7687	0.049*	
C21	0.9395 (4)	0.46935 (9)	0.79385 (17)	0.0423 (5)	
C22	1.1471 (6)	0.48056 (12)	0.8727 (2)	0.0678 (8)	
H22	1.2159	0.515	0.8798	0.081*	
C23	1.2529 (6)	0.44077 (16)	0.9412 (3)	0.0881 (10)	
H23	1.395	0.4487	0.9931	0.106*	
C24	1.1543 (7)	0.39089 (16)	0.9341 (3)	0.0861 (11)	
H24	1.2225	0.3648	0.9826	0.103*	
C25	0.9545 (8)	0.37876 (13)	0.8557 (3)	0.0920 (11)	
H25	0.8906	0.344	0.8487	0.11*	
C26	0.8446 (6)	0.41794 (11)	0.7858 (2)	0.0704 (8)	
H26	0.7056	0.4093	0.733	0.085*	
N1	1.0341 (4)	0.61630 (7)	0.72100 (16)	0.0442 (5)	
N2	1.0180 (3)	0.53247 (7)	0.64038 (13)	0.0345 (4)	
O1	1.4457 (3)	0.63131 (7)	0.81329 (15)	0.0619 (5)	
S1	1.38677 (11)	0.59942 (2)	0.55656 (5)	0.0483 (2)	
H1N	0.855 (2)	0.6203 (9)	0.7200 (19)	0.053 (7)*	

### Atomic displacement parameters (Å^2^)

**Table:**

	*U*^11^	*U*^22^	*U*^33^	*U*^12^	*U*^13^	*U*^23^
C1	0.0269 (10)	0.0383 (12)	0.0413 (12)	0.0072 (9)	−0.0054 (8)	−0.0019 (9)
C3	0.0358 (11)	0.0373 (12)	0.0398 (12)	0.0061 (9)	−0.0028 (9)	−0.0038 (9)
C10	0.0430 (13)	0.0431 (13)	0.0515 (14)	−0.0053 (10)	0.0050 (10)	−0.0062 (11)
C11	0.0496 (14)	0.0408 (14)	0.0692 (17)	−0.0103 (12)	0.0197 (12)	−0.0113 (12)
C12A	0.068 (5)	0.037 (6)	0.092 (5)	0.006 (4)	0.017 (4)	−0.010 (4)
C13A	0.081 (5)	0.040 (4)	0.109 (9)	0.008 (4)	0.015 (5)	−0.023 (4)
C14A	0.074 (7)	0.047 (4)	0.114 (9)	−0.003 (4)	0.021 (6)	−0.036 (5)
C15A	0.088 (5)	0.063 (5)	0.083 (5)	−0.008 (4)	0.018 (4)	−0.040 (4)
C16A	0.068 (4)	0.055 (5)	0.071 (4)	−0.005 (3)	0.010 (3)	−0.029 (3)
C12B	0.086 (12)	0.035 (9)	0.108 (14)	−0.010 (8)	0.051 (11)	−0.003 (9)
C13B	0.113 (13)	0.068 (9)	0.112 (15)	−0.028 (9)	0.029 (11)	−0.039 (11)
C14B	0.094 (13)	0.060 (10)	0.095 (15)	−0.015 (10)	0.016 (11)	−0.033 (10)
C15B	0.089 (18)	0.060 (13)	0.090 (12)	−0.012 (11)	0.015 (12)	−0.014 (11)
C16B	0.071 (12)	0.053 (11)	0.103 (11)	−0.020 (9)	0.024 (8)	−0.012 (9)
C20	0.0320 (10)	0.0471 (13)	0.0437 (12)	−0.0021 (10)	0.0012 (9)	−0.0005 (10)
C21	0.0395 (11)	0.0475 (13)	0.0403 (12)	0.0028 (10)	0.0078 (9)	0.0014 (10)
C22	0.0624 (16)	0.0777 (19)	0.0625 (17)	−0.0047 (14)	−0.0143 (13)	0.0092 (15)
C23	0.070 (2)	0.123 (3)	0.071 (2)	0.007 (2)	−0.0164 (15)	0.031 (2)
C24	0.082 (2)	0.102 (3)	0.076 (2)	0.029 (2)	0.0143 (18)	0.040 (2)
C25	0.122 (3)	0.0551 (19)	0.099 (3)	0.005 (2)	0.015 (2)	0.0219 (18)
C26	0.083 (2)	0.0545 (17)	0.073 (2)	−0.0065 (15)	−0.0062 (16)	0.0080 (14)
N1	0.0290 (9)	0.0431 (11)	0.0605 (12)	0.0037 (8)	0.0001 (8)	−0.0168 (9)
N2	0.0309 (8)	0.0370 (10)	0.0355 (9)	0.0025 (7)	0.0006 (7)	−0.0037 (7)
O1	0.0418 (9)	0.0742 (12)	0.0691 (12)	0.0019 (9)	−0.0104 (8)	−0.0170 (9)
S1	0.0406 (3)	0.0480 (4)	0.0568 (4)	0.0003 (3)	0.0084 (3)	0.0033 (3)

### Geometric parameters (Å, º)

**Table:**

C1—N2	1.325 (3)	C12B—H12B	0.93
C1—N1	1.408 (3)	C13B—C14B	1.361 (15)
C1—S1	1.673 (2)	C13B—H13B	0.93
C3—N2	1.460 (2)	C14B—C15B	1.374 (17)
C3—C3^i^	1.523 (4)	C14B—H14B	0.93
C3—H3A	0.97	C15B—C16B	1.379 (16)
C3—H3B	0.97	C15B—H15B	0.93
C10—O1	1.227 (3)	C16B—H16B	0.93
C10—N1	1.370 (3)	C20—N2	1.476 (3)
C10—C11	1.474 (3)	C20—C21	1.504 (3)
C11—C16B	1.362 (16)	C20—H20A	0.97
C11—C12A	1.386 (7)	C20—H20B	0.97
C11—C12B	1.387 (18)	C21—C26	1.373 (3)
C11—C16A	1.403 (8)	C21—C22	1.381 (3)
C12A—C13A	1.395 (10)	C22—C23	1.382 (4)
C12A—H12A	0.93	C22—H22	0.93
C13A—C14A	1.371 (9)	C23—C24	1.343 (5)
C13A—H13A	0.93	C23—H23	0.93
C14A—C15A	1.394 (9)	C24—C25	1.356 (4)
C14A—H14A	0.93	C24—H24	0.93
C15A—C16A	1.399 (9)	C25—C26	1.388 (4)
C15A—H15A	0.93	C25—H25	0.93
C16A—H16A	0.93	C26—H26	0.93
C12B—C13B	1.388 (17)	N1—H1N	0.861 (10)
			
N2—C1—N1	116.24 (18)	C13B—C14B—C15B	123 (2)
N2—C1—S1	124.30 (16)	C13B—C14B—H14B	118.5
N1—C1—S1	119.40 (16)	C15B—C14B—H14B	118.5
N2—C3—C3^i^	110.9 (2)	C14B—C15B—C16B	118 (2)
N2—C3—H3A	109.5	C14B—C15B—H15B	120.9
C3^i^—C3—H3A	109.5	C16B—C15B—H15B	120.9
N2—C3—H3B	109.5	C11—C16B—C15B	124 (2)
C3^i^—C3—H3B	109.5	C11—C16B—H16B	117.9
H3A—C3—H3B	108	C15B—C16B—H16B	117.9
O1—C10—N1	121.0 (2)	N2—C20—C21	111.86 (16)
O1—C10—C11	122.1 (2)	N2—C20—H20A	109.2
N1—C10—C11	116.9 (2)	C21—C20—H20A	109.2
C16B—C11—C12B	112 (2)	N2—C20—H20B	109.2
C12A—C11—C16A	121.1 (8)	C21—C20—H20B	109.2
C16B—C11—C10	132.2 (14)	H20A—C20—H20B	107.9
C12A—C11—C10	124.0 (7)	C26—C21—C22	118.0 (2)
C12B—C11—C10	115.6 (18)	C26—C21—C20	121.4 (2)
C16A—C11—C10	114.7 (6)	C22—C21—C20	120.5 (2)
C11—C12A—C13A	118.2 (10)	C21—C22—C23	120.2 (3)
C11—C12A—H12A	120.9	C21—C22—H22	119.9
C13A—C12A—H12A	120.9	C23—C22—H22	119.9
C14A—C13A—C12A	121.7 (8)	C24—C23—C22	121.2 (3)
C14A—C13A—H13A	119.1	C24—C23—H23	119.4
C12A—C13A—H13A	119.1	C22—C23—H23	119.4
C13A—C14A—C15A	120.3 (7)	C23—C24—C25	119.5 (3)
C13A—C14A—H14A	119.9	C23—C24—H24	120.2
C15A—C14A—H14A	119.9	C25—C24—H24	120.2
C14A—C15A—C16A	119.3 (8)	C24—C25—C26	120.4 (3)
C14A—C15A—H15A	120.3	C24—C25—H25	119.8
C16A—C15A—H15A	120.3	C26—C25—H25	119.8
C15A—C16A—C11	119.4 (8)	C21—C26—C25	120.5 (3)
C15A—C16A—H16A	120.3	C21—C26—H26	119.7
C11—C16A—H16A	120.3	C25—C26—H26	119.7
C11—C12B—C13B	129 (4)	C10—N1—C1	122.61 (17)
C11—C12B—H12B	115.6	C10—N1—H1N	121.7 (16)
C13B—C12B—H12B	115.6	C1—N1—H1N	115.6 (16)
C14B—C13B—C12B	113 (3)	C1—N2—C3	120.25 (17)
C14B—C13B—H13B	123.5	C1—N2—C20	125.18 (17)
C12B—C13B—H13B	123.5	C3—N2—C20	114.56 (16)
			
O1—C10—C1—S1	−62.55 (17)	S1—C1—N1—C10	−49.1 (3)
O1—C10—N1—C1	−24.3 (3)		

Symmetry code: (i) −*x*+2, −*y*+1, −*z*+1.

### Hydrogen-bond geometry (Å, º)

**Table:**

*D*—H···*A*	*D*—H	H···*A*	*D*···*A*	*D*—H···*A*
N1—H1*N*···O1^ii^	0.86 (1)	2.30 (1)	3.073 (2)	150 (2)
N1—H1*N*···S1^ii^	0.86 (1)	2.98 (2)	3.647 (2)	136 (2)

Symmetry code: (ii) *x*−1, *y*, *z*.
